# Variation in Attitudes to Native Kidney Biopsy Practice in the United States

**DOI:** 10.1016/j.xkme.2025.101174

**Published:** 2025-11-07

**Authors:** Michael P. Toal, Christopher J. Hill, Michael P. Quinn, Emily P. McQuarrie, Charuhas V. Thakar, Ciaran O’Neill, Alexander P. Maxwell

**Affiliations:** 1 Centre for Public Health, Grosvenor Road, Queen’s University Belfast, Belfast, Northern Ireland; 2 Regional Centre for Nephrology and Transplantation, Belfast City Hospital, Belfast, Northern Ireland; 3 Glasgow Renal and Transplant Unit, Queen Elizabeth University Hospital, Glasgow, Scotland; 4 Wellcome-Wolfson Institute for Experimental Medicine, Queen’s University Belfast, Belfast, Northern Ireland; 5 Division of Nephrology and Hypertension, University of Cincinnati, Cincinnati, OH

**Keywords:** Kidney biopsy pathology, kidney biopsy questionnaire

## Abstract

**Rationale & Objective:**

There is substantial variation in kidney biopsy practices within and between countries; however, the reasons for this are unclear due to limited research among large diverse populations. The aim of this study was to explore variations in attitude to the indications and contraindications for native kidney biopsy in the United States (US).

**Study Design:**

A case-vignette questionnaire was developed. A propensity-to-biopsy score (0-44) was generated from responses to indications and contraindications, with a higher score indicating an increased likelihood to recommend biopsy. Dissemination of the questionnaire occurred by email, social media, and the National Kidney Foundation.

**Setting & Participants:**

A total of 295 nephrologists/fellows from 43 states within the US participated.

**Exposure:**

All participants completed an identical questionnaire on kidney biopsy practice.

**Outcomes:**

Responses were collected on indications, contraindications, and attitudes to biopsy.

**Analytical Approach:**

Anonymized IP addresses were collected for comparison between US states. Data were also collected on the demographics of the individual and the type of institution in which the doctor was based.

**Results:**

In an adjusted multiple linear regression analysis, higher propensity-to-biopsy scores were demonstrated in US clinicians who were male, younger and more frequent performers of kidney biopsy (*P* = 0.05). There were significant differences between the 18 US states with 5 or more participants (*P* < 0.001) with the mean propensity-to-biopsy score ranging from 20.3 (Wisconsin) to 29.2 (New Jersey and Virginia). Increased biopsy propensity was also observed in US states with higher nephrologist density and lower statewide deprivation (*P* = 0.006).

**Limitations:**

The condensed clinical scenarios may not accurately replicate real-world cases, and clinicians opted in often using social media, so generalizability is limited.

**Conclusions:**

Attitudes to kidney biopsy practice in the US are highly variable, and clinician or institutional characteristics do not fully explain these discrepancies. Further research is required to understand the factors that influence clinical decision making.

## Plain-Language Summary

A kidney biopsy can be used to diagnose particular types of kidney disease; however, it exposes patients to a small risk of serious bleeding. A questionnaire was completed by kidney specialist doctors in the United States to determine when this test is best used. There were significant differences in attitudes between these doctors based on their sex, age, and state. States with less poverty and more specialist doctors were shown to have greater willingness to recommend biopsy. Doctors surveyed in the United States were more likely to recommend biopsy than doctors in the United Kingdom. The study is limited, as it does not measure real-world practice, and study participants were not reflective of the whole profession.

Kidney biopsy has been used in nephrology since the 1950s for the diagnosis of kidney disease and to inform management.^1^^,^^2^ Despite it being accepted as the gold standard diagnostic test, widespread variation in kidney biopsy practices is recognized both across centers and between countries.^3^ There is limited understanding of the causes and effects of these variations within the currently available literature, with studies restricted to clinician surveys of single countries with limited participant numbers.^4^^,^^5^

Advances in precision medicine are improving our understanding of the pathogenesis of kidney disease at the molecular level. Initiatives like the Kidney Precision Medicine Project use biopsy samples for advanced proteomic and genomic analysis to identify novel markers of risk.^6^ Multiple new treatments for biopsy-proven kidney disease have been evaluated through randomized controlled trials, and these novel therapies are entering clinical practice.^7^, ^8^, ^9^, ^10^ This requires a more consistent standard of when diagnostic tools such as kidney biopsy should be applied so that patients can access effective treatment in a timely manner.

The practice of kidney biopsy varies internationally and is traditionally under the remit of nephrology and interventional radiology. In the United States (US), nephrologists are increasingly referring to radiologists rather than performing this procedure themselves, and fellows are not required to be competent in performing kidney biopsy for certification.^11^^,^^12^ This could lead to a cultural distancing of nephrologists in the practice and value of a kidney biopsy.

One of the reasons nephrologists may be cautious about kidney biopsy is the recognized small risk of complications. Serious complications can arise, particularly relating to bleeding.^1^ Persistent hemorrhage may require radiological embolization in around 1 in 300 biopsy procedures, and death linked to kidney biopsy has been reported in around 1 in 2000 procedures.^13^, ^14^, ^15^, ^16^

The decision to perform a kidney biopsy remains subjective and dependent on multiple factors unique to each patient. Each clinician across the US and internationally operates within a different context of resources, therefore decisions may be subject to these constraints.^4^^,^^5^

### Objective

This study examines the hypothesis that attitudes to kidney biopsy vary among nephrologists in the US.

## Methods

Findings are described in line with the checklist for reporting results of internet e-surveys.^17^ A detailed explanation of methods have been described in a previous paper^18^ but are briefly summarized below.

### Questionnaire Design

The study was part of a larger international study of attitudes to kidney biopsy.^18^ Clinicians who were eligible for inclusion here were physicians practicing in the US who specialized in adult nephrology. A case-vignette online questionnaire was designed to explore the reasoning behind clinical practice variation, which is detailed in the supplementary material (Item S1). The first section focused on collecting anonymized demographic details of the individual and their institution. The second section focused on decisions and attitudes. Seven scenarios of case vignettes were presented for the physician to decide if a kidney biopsy was required for each patient on a 5-point scale from definitely yes to definitely no. For contraindications, respondents were asked to define the tolerable limits of laboratory results, clinical observations, and prescribed drugs that may contraindicate biopsy. Attitudes and previously cited barriers were explored in the final part of the questionnaire.^19^

### Propensity-to-Biopsy Score

Responses to 11 questions in the indications and contraindications section were coded from 0-4. For the case vignettes, if the respondent selected definitely yes, this was scored as 4 points, with decreasing values for each option to definitely no, which scored 0 points. For contraindications, the closest option to a normal value (eg, platelet count = 150 × 10^9^) was scored as 0, whereas the selected option of proceeding to biopsy regardless of the value (eg, no minimum level) was scored as 4.

This scoring system generated a total propensity-to-biopsy score from 0-44 for each clinician who completed the entire questionnaire, with a higher score signifying the clinician was more likely to recommend biopsy.

### Patient and Public Engagement Summary

Ethical approval for this project was granted by the research ethics committee of Queen’s University Belfast and all participants indicated their consent and eligibility before entering the questionnaire. The questionnaire was co-designed with patient participation from 10 patients who had underwent a total of 23 kidney biopsies from across the United Kingdom (UK). This study explored the barriers and experience of kidney biopsy from a patient’s perspective,^20^ which facilitated further study of the physician’s perspective.

### Questionnaire Administration

The survey was piloted first within the research team and then in one UK region before wider international dissemination. Participants in the US were recruited through an anonymized open web address, which was shared through email lists of nephrology societies, including the National Kidney Foundation, and on social media.

The questionnaire was hosted on Qualtrics XM, which collected anonymized IP addresses for state-level analysis, based on the assumption that the questionnaire was completed in the participant’s state of clinical practice. This software prevented multiple entries and used a completely automated public Turing test to tell computers and humans apart feature to safeguard against automated entries. International recruitment occurred from August 29, 2023, to January 14, 2024. Because of the nature of US distribution through social media and the National Kidney Foundation, it is difficult to estimate response rates. Within regions of the UK and Ireland where the survey was also undertaken, over 50% of eligible clinicians participated.

### Analysis

All clinicians who met the inclusion criteria and answered one or more questions were contained in the final dataset; however, the propensity-to-biopsy score was only counted if all relevant questions were answered.

To help contextualize responses, data were obtained on nephrologist density per 100,000 adults by state.^21^ The US Census data were used to arrange states by deprivation,^22^ which were grouped into quintiles with similar numbers of clinicians. Analysis by state focused on states with 5 or more participants. For international context, US responses were compared with the country with the second highest national participation, the UK.

The complete dataset was analyzed in Stata 17 (StataCorp). Aggregate results were expressed as means if normally distributed or medians if skewed. Discrete variables were reported by frequency. An unpaired *t* test was used to compare continuous variables between 2 groups and ANOVA with Bonferroni adjustment in the setting of 3 or more groups. A severe complication was defined as one that required an intervention of embolization, surgery or that resulted in death. An adjusted multiple linear regression model was applied to ordinal or continuous demographic categories to assess the impact on propensity score. Significance levels were tested at the 5% level (*P* < 0.05).

## Results

### National Demographics

A total of 295 US clinicians participated in the study. Findings from the entire international cohort are detailed in another article.^18^ The US clinician and institutional demographics are summarized in Table 1. The entire questionnaire was completed by 92.2% of US participants. A broad range of age groups were represented; however, only 26% of respondents identified as female, which is consistent with the current US workforce.^23^ About 64.7% of the US clinicians had performed at least one kidney biopsy in the previous year; however, for 66.8% of participating clinicians, the usual operator in their institution was a radiology specialist. About 46.2% of clinicians reported that the most significant biopsy complication they had ever encountered was one requiring an invasive intervention or causing death, classed as a severe complication in this study.

**Table 1:** Individual and Institutional Demographic Details of Clinicians

Variable	Frequency	%
Questionnaire completed		
Yes	272	92.2
No	23	7.8
Route of completion		
Email link	135	45.8
Social media	160	54.2
Sex		
Male	211	72.3
Female	76	26.0
Prefer not to say	4	1.4
Nonbinary/third gender	1	0.3
Age, y		
20-29	2	0.7
30-39	102	34.6
40-49	72	24.4
50-59	67	22.7
60 or over	52	17.6
Current job title		
Trainee/fellow	18	6.1
Associate specialist/specialty doctor	11	3.8
Consultant/attending physician	235	80.2
Clinical director or professor	28	9.6
Other	1	0.3
Estimated No. of biopsies performed in last year		
0	104	35.3
1-5	54	18.3
5-20	103	34.9
20-50	24	8.1
50 or more	10	3.4
Most significant complication encountered		
Nonsevere	156	54.8
None	36	12.4
Macroscopic hematuria	64	22.1
Hematoma	4	1.4
Blood transfusion	50	17.2
Nonrenal organ injury	1	0.3
Other	1	0.3
Severe	134	46.2
Radiological embolization	99	34.1
Emergency surgery	2	0.7
Nephrectomy	11	3.8
Death	22	7.6
Health care system		
Public	79	27.2
Private	134	46.2
Public and private	67	23.1
Not sure	10	3.5
Main place of work		
Independent clinic	21	7.3
Rural or district general hospital	16	5.5
Suburban hospital	81	28.0
University/Urban/Military hospital	170	58.8
Other	1	0.4
Typical time from referral to biopsy by radiologist		
Same day	10	3.5
Within 1 week	171	59.0
Within 1 month	84	29.0
Beyond 1 month	2	0.7
Unsure	23	7.9
Most frequent performer of kidney biopsy at institution		
Nephrologist	71	24.6
Radiologist	187	64.7
Nephrology trainee/fellow	24	8.3
Radiology trainee/fellow	6	2.1
Not sure	1	0.4
Observation time after kidney biopsy		
Less than 4 h	49	17.0
4-8 h	173	59.9
8-24 h	58	20.1
Beyond 24 h	5	1.7
Unsure	4	1.4

### State-Level Comparison

Forty-three US states were represented in this study and in 18 states, 5 or more clinicians contributed (Table 2). For comparisons, only US states with at least 5 clinicians were included in statistical analysis. To investigate the social and economic influences on attitudes to practice, data were obtained on the relative deprivation of each state,^22^ which was then divided into quintiles to provide similar sized groups for each quintile of deprivation (55-63 clinicians each).

**Table 2:** US State-Level Data on Participants

US State	No. of Participants	Mean Propensity-to-Biopsy Score	Deprivation Quintile	Nephrologists/100,000 Adults
Alabama	2	22	1	3
Alaska	1	27	3	0.8
Arizona	4	27.5	2	2.9
Arkansas	1	27	1	2.3
California	26	23.5	3	3.1
Colorado	3	23.7	5	2.6
Connecticut	1	30	4	4.6
Delaware	0	N/A	N/A	4
Florida	11	21.4	2	3
Georgia	6	25	2	3.3
Hawaii	3	25	4	2.2
Idaho	0	N/A	N/A	1.6
Illinois	22	25.7	4	3.1
Indiana	1	28	3	3.2
Iowa	2	22	4	2
Kansas	1	17	4	2.6
Kentucky	2	22.5	1	2.8
Louisiana	4	23.3	1	4
Maine	1	28	4	2.5
Maryland	6	28	4	4.4
Massachusetts	11	25.9	5	5.3
Michigan	8	24.5	3	3.2
Minnesota	3	24.3	5	3.5
Mississippi	5	22.3	1	3.1
Missouri	6	24	3	3.2
Montana	0	N/A	N/A	1
Nebraska	2	27	5	2.3
Nevada	3	21	2	2.3
New Hampshire	1	24	5	1.8
New Jersey	6	29.2	5	4
New Mexico	1	29	1	2.1
New York	25	25.5	2	4.7
North Carolina	9	24.3	2	3.4
North Dakota	0	N/A	N/A	2.7
Ohio	12	24	3	3
Oklahoma	2	25	1	2.2
Oregon	2	22	4	2.2
Pennsylvania	12	26.8	4	4
Rhode Island	1	26	4	4.2
South Carolina	7	20.4	1	3.1
South Dakota	0	N/A	N/A	3.6
Tennessee	8	25.4	3	3.4
Texas	29	23.3	1	3.3
Utah	1	28	5	1.7
Vermont	0	N/A	N/A	2
Virginia	6	29.2	4	3
Washington	27	24.2	5	2.7
West Virginia	0	N/A	N/A	2.7
Wisconsin	7	20.3	5	2.5
Wyoming	0	N/A	N/A	0.9
District of Columbia	2	20.5	1	N/A

*Note:* Missing data reported as N/A. Deprivation quintiles: 1 = most deprived, 5 = least deprived.

**Figure 1:**
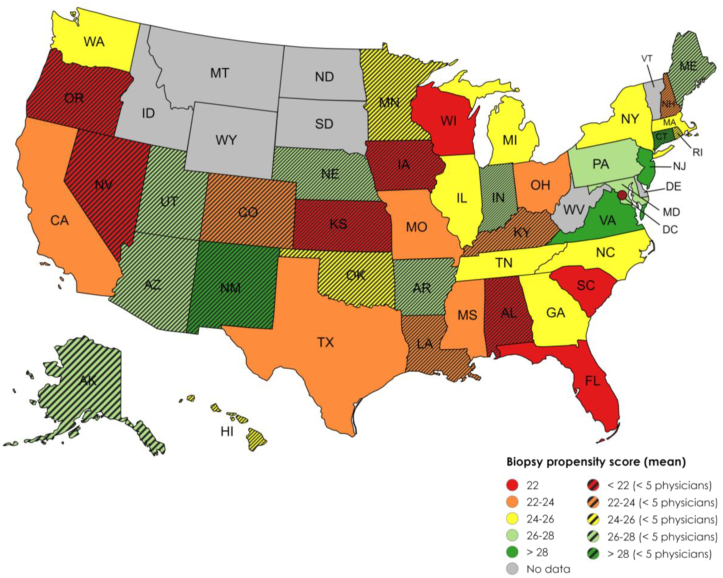
Mean propensity-to-biopsy score divided into the state where clinicians completed the questionnaire. A dark green color is associated with the highest propensity score and red with the lowest. Striped states had less than 5 participants and were not included in statistical analysis. Graphic courtesy of mapchart.net.

In states with at least 5 participants, the highest propensity-to-biopsy score was observed in clinicians from New Jersey and Virginia (29.2), and the lowest was in Wisconsin (20.3). A breakdown of the mean propensity score by state is represented in Fig 1. The state with the highest nephrologist density per 100,000 adults was Massachusetts at 5.3 per 100,000 adults, and the lowest nephrologist density was Alaska at 0.8 per 100,000 adults.

### Analyzing Attitudes to Practice

A multiple linear regression model was used to investigate the association between individual, institutional, and state characteristics and how this impacted on propensity-to-biopsy scores. All variables were included in the first model, and then regression with backward elimination was used to sequentially remove nonsignificant variables. The variables that remained significant after adjustment are described in Table 3.

**Table 3:** Adjusted Multiple Linear Regression Table of the Effect of Demographic Variables on Propensity-to-Biopsy Score

Variable	Coefficient	SE	Significance	95%CI·Lower	95% CI Upper
Sex (=female)	−1.240	0.624	0.048	−2.469	−0.012
Age (by increasing age group)	−0.596	0.246	0.016	−1.080	−0.111
Biopsy frequency (by increasing group)	+0.555	0.247	0.026	+0.068	+1.042
Deprivation quintile	+0.532	0.191	0.006	+0.155	+0.909
Nephrologist density/100,000 population	+1.146	0.348	0.001	+0.460	+1.832

*Note:* n = 257, F = 6.50. Adjusted R^2^ = 0.097.

A number of variables were significantly associated with increased propensity-to-biopsy scores. Male physicians, those of younger age, and more regular performers of kidney biopsy were all significantly associated with increased biopsy propensity scores, after adjustment for other factors (*P* = 0.05). These variables demonstrated a similar association in the full international cohort.

Nephrologists from less deprived states reported a greater willingness to biopsy, as did those from states with higher numbers of nephrologists per head of the adult population. While the overall regression was significant as evidenced by the F-statistic, collectively the explanatory variables used only accounted for 10% of the variation in scores; therefore, 90% of the variation in scores remains unexplained by the variables in this model.

### Comparing US Practice to the UK

To examine US practice in a global context, US responses were compared to the second largest national group in this study, the UK (n = 213). Although these countries are aligned in many geopolitical and social issues, their health care systems differ in terms of funding (a lower proportion of Gross Domestic Product is spent on health care in the UK), population, and provision of care (the UK health care is free at the point of need). Among respondents to this survey, only 27.2% of US clinicians worked in a solely publicly funded service, in contrast to 98% of UK clinicians. We hypothesized that economic factors for clinicians would differ between clinicians in these 2 nations and might influence attitudes. Additionally, there are significant differences in clinical practice. 88% of UK participants indicated that the usual biopsy operator was a nephrologist or nephrology fellow at their institution, compared to only 33% of US clinicians, in which most procedures were performed by a radiologist (*P* < 0.001).

The mean propensity-to-biopsy score was significantly higher in the US (24.5) compared with the UK (23.5) (*P* = 0.01). The most significant barriers to biopsy access differed between respondents in the 2 countries (Table 4). The most significant barrier for US clinicians was time constraints, whereas this was deemed to be the least significant factor in the UK. By contrast, the least significant barrier reported by US clinicians was the risk of complications, which was deemed to be the most significant barrier by UK clinicians (*P* < 0.001). Therefore, in this study it appeared that perceptions on barriers to access were highly contingent on what the typical practice was in that country. US clinicians rated the barriers to biopsy access as more significant than respondents from the UK (*P* < 0.001).

**Table 4:** Comparison of Perceived Barriers to Kidney Biopsy Between US and UK Clinicians

United States	United Kingdom
1. Time constraints	1. Risk of complications
2. Staff availability	2. Staff availability
3. Room and equipment availability	3. Room and equipment availability
4. Risk of complications	4. Time constraints

*Note:* Respondents were asked to rate the significance of each factor from 1 (least significant) to 5 (most significant). The potential barriers are ranked for each country in order from most to least significant.

## Discussion

### General Interpretation

This study suggests that there are variations in attitudes to kidney biopsy practice across the US. Males, younger physicians, and more frequent biopsy operators were found to have an increased propensity to recommend kidney biopsy, as were individuals working in states with higher nephrologist coverage and economic affluence. While the significance of practitioner density and deprivation are interesting, caution is warranted given the potential for ecological fallacy, that is, that inferences are made about an individual based on their membership of a group. Therefore, we cannot assume that a nephrologist in Massachusetts would make a different decision to their colleague in Oklahoma in a given clinical scenario, particularly as the variables in the regression model could only account for 10% of the variation in scores.

It is difficult to explain the variations in biopsy rates between states, but it may be that where there is a higher density of nephrologists, there is a higher capacity to provide services such as a kidney biopsy. However, these inferences are speculative and require investigation beyond the scope of this study.

This study also revealed some interesting findings around postbiopsy observation periods, where again there were variations in practice; however, most centers observed patients for 8 hours or less postprocedure. This has economic consequences for health care delivery, as patients will not need an overnight admission to hospital for observation after a routine kidney biopsy.

It is notable that US clinicians report different barriers to kidney biopsy than their UK colleagues. US-based nephrologists reported that time constraints were the most significant barrier to biopsy, whereas UK clinicians reported the risk of complications as the predominant limitation. There are relevant differences in health care systems and clinical practice that have been outlined. Potential explanations for these disparities could be that US clinicians who bill for each service may feel the time taken to organize and perform a biopsy may not be a cost-effective use of time compared with other clinical activities. Alternatively, as US nephrologists often delegate this procedure to radiologist colleagues rather than performing biopsies themselves, the responsibility of exposing patients to the potential risks is shared with the operator performing the procedure rather than being borne solely by the nephrologist. However, a circumspect approach is best applied in this instance, as these inferences are beyond the scope of this study.

A Scottish study demonstrated that greater socioeconomic deprivation was associated with higher kidney biopsy rates^24^; however, in the UK, health care is free at the point of access for all citizens in a nationalized public health system, therefore, these findings may not be directly translatable to the US. The privatized US health care system and greater cultural and racial diversity could indeed widen these gaps in health care attainment, fortifying the importance of understanding the barriers to achieving equitable health care delivery. Not all Americans have health insurance, which may affect access to a kidney biopsy. One of the limitations of a questionnaire is that although the differences are statistically significant, it is unclear whether they truly reflect clinically significant practice variations resulting in higher or lower rates of biopsies in the future. There may be differences in how someone might answer this survey based on their location, which may not be reflective of actual clinical practice.

Amodou et al^25^ interviewed 20 nephrologists to understand their attitudes to practice. In this study, nephrologists who performed their own biopsies reported that they believed this heuristic lowered their threshold to order this procedure, which is consistent with our findings.

### Limitations

This study has potential limitations. This is an opt-in online questionnaire to investigate attitudes to practice and is more likely to appeal to clinicians with an interest in kidney biopsy, who may be disproportionately represented in a small sample size relative to the total eligible population. The use of social media resulted in a relatively young study population, which may not be generalizable to the entire profession.

The area-based characteristics investigated are reliant on previously collected records, which may not be entirely accurate or contemporaneous and will not reflect the granularity of population or practice within each state. The clinical vignettes are insufficient in detail to reflect all of the considerations, which may be required to make a decision on kidney biopsy. The vignettes are condensed clinical scenarios to promote participant engagement to help in determining the predominant driving and limiting factors of practice.

### Strengths

The study also has significant strengths. In addition to being the largest international study of kidney biopsy practice, the high uptake from US clinicians at various stages of their careers, from fellowship to extended independent practice, has also made it the largest study of US attitudes that the authors are aware of. Additionally, the representation from 43 states increases generalizability at a national level. Furthermore, the opportunity to compare the findings internationally strengthens the evaluation.

### Implications for Practice

This study demonstrates that there is variation in attitudes to kidney biopsy practice when nephrologists are confronted with identical clinical scenarios. Most of the variation demonstrated remains unexplained by demographics of either the individual participant or their working environment. Additionally, it also remains unclear if the variation in attitudes reported translates into measurable differences in clinical practice, which could have implications for patient access. Such studies can be used as a feedback mechanism to inform practitioners, providing insight into the real and perceived variations that can in turn facilitate standardization of practices.

## Conclusion

Attitudes to kidney biopsy practice were found to be variable across the US and were associated with individual and area-based factors. Strikingly, only 10% of the variation in propensity-to-biopsy scores could be explained in terms of observable characteristics. We propose that a study such as ours will inform physicians of key parameters that could be overcome in reducing variations in practice. Further prospective monitoring would be required to understand if such efforts affect uniformity in practice.

## Supplementary Material

Supplementary File (PDF) Item S1.
