# Supplementary material for: Variation in Attitudes to Native Kidney Biopsy Practice in the United States
Source: Kidney Med. 2025 Nov 7;8(1):101174. doi: 10.1016/j.xkme.2025.101174 (PMC12771087; doi:10.1016/j.xkme.2025.101174)
Supplement: Supplementary File (PDF) — Item S1. [file mmc1.pdf]

Item S1. Electronic Questionnaire Instrument

Start of Block: Demographic information

Consent Thank you for taking this questionnaire. You will be contributing to a PhD study of international renal biopsy practice. All responses will be anonymous. Please check eligibility criteria before continuing.

Estimated completion time: Less than 5 minutes

- ○ I am a medical doctor specialising in Adult Nephrology/Renal Medicine. I consent to participate
- ○ I do not meet the inclusion criteria and am ineligible for this study

Q2 Please verify you are a human to continue

Page Break

Q1 What age are you?

- ○ 20-29
- ○ 30-39
- ○ 40-49
- ○ 50-59
- ○ 60 or over

Q2 What is your sex?

- ○ Male
- ○ Female
- ○ Non-binary / third gender
- ○ Prefer not to say

Q3 What is your current job title?

- ○ Clinical Director or Lead
- ○ Consultant or Attending Physician
- ○ Associate Specialist or Specialty Doctor
- ○ Trainee or Fellow
- ○ Other (please specify) ____

Q4 How many renal biopsies have you performed in the last year?

- ○ 0
- ○ 1-5
- ○ 5-20
- ○ 20-50
- ○ 50+

Page Break

Q4b When did you last perform a renal biopsy?

- ○ Within 2 years
- ○ Within 5 years
- ○ More than 5 years ago
- ○ I have never performed a renal biopsy

Q5 What is your most significant renal biopsy complication where you have been involved in any capacity?

- ○ Death
- ○ Nephrectomy
- ○ Embolisation
- ○ Blood transfusion
- ○ Haematuria
- ○ Other (please specify) ____
- ○ No complications encountered

End of Block: Demographic information

Start of Block: Environment

Q1 What country do you work in?

Click box to type

▼ England ... Zimbabwe

Q2 Which sector do you work in?

- ◯ Public healthcare system
- ◯ Private healthcare system
- ◯ Both public and private healthcare
- ◯ Not sure

Q3 Where is your main place of work?

- ◯ Urban hospital
- ◯ Suburban hospital
- ◯ Rural Hospital
- ◯ Independent Clinic
- ◯ Other (please specify) ____

Q4 When referred to Interventional Radiology, what is the typical waiting time for a renal biopsy?

- ◯ Same day
- ◯ Within one week
- ◯ Within one month
- ◯ Beyond one month
- ◯ Unsure

Q5 In your nephrology department, who would perform the most renal biopsies?

- ○ Nephrologist
- ○ Radiologist
- ○ Nephrology trainee/fellow
- ○ Radiology trainee/fellow
- ○ Not sure

Q5b Who would most often be responsible for supervision of renal biopsies?

- ○ Nephrologist
- ○ Radiologist
- ○ Not sure

Page Break

Q6 At your institution, how long are patients observed for after an uncomplicated renal biopsy?

- ○ Less than 4 hours
- ○ 4-8 hours
- ○ 8-24 hours
- ○ Beyond 24 hours
- ○ Not sure

Page Break

6b Prior to discharge after renal biopsy, what imaging is routinely performed?

- ○ Ultrasound
- ○ CT scan
- ○ MRI scan
- ○ Other imaging
- ○ No routine imaging

End of Block: Environment

Start of Block: Indications. V2

Q1 In your opinion- is a renal biopsy required for an **adult** in the **first detection** of an **unexplained nephrotic syndrome of proteinuria 4g/day, peripheral oedema and eGFR > 60** ml/min/1.73m2?

- ○ Definitely yes
- ○ Probably yes
- ○ Unsure
- ○ Probably not
- ○ Definitely not

Page Break

Q2 In your opinion, is a renal biopsy required for an adult in the **first detection** of unexplained **non-visible haematuria, 2g/day of proteinuria and eGFR 40?**

- Definitely yes
- Probably yes
- Unsure
- Probably not
- Definitely not

Page Break

Q3 In your opinion, is a renal biopsy required for an adult in the **first detection** of unexplained **non-visible haematuria, 2g/day of proteinuria and eGFR 20** with **normal kidney appearances on ultrasound?**

- ◯ Definitely yes
- ◯ Probably yes
- ◯ Unsure
- ◯ Probably not
- ◯ Definitely not

Page Break

Q4 In your opinion, is a renal biopsy required for an adult in the **first detection** of unexplained **non-visible haematuria, 2g/day of proteinuria and eGFR 20** with **reduced kidney size on ultrasound?**

- ○ Definitely yes
- ○ Probably yes
- ○ Unsure
- ○ Probably not
- ○ Definitely not

Page Break

Q5 In your opinion, is a renal biopsy required for an adult with an **unexplained rise in proteinuria from 0.5 to 2g/day in one year with an eGFR > 60**

- ○ Definitely yes
- ○ Probably yes
- ○ Unsure
- ○ Probably not
- ○ Definitely not

Page Break

Q6 In your opinion, is a renal biopsy required for an adult with an **unexplained fall in eGFR from 55 to 40 in one year with proteinuria stable at 0.5g/day?**

- ○ Definitely yes
- ○ Probably yes
- ○ Unsure
- ○ Probably not
- ○ Definitely not

Page Break

Q7 In your opinion, is a renal biopsy required for an adult with an **unexplained fall in eGFR from 55 to 40 AND rise in proteinuria from 0.5 to 2 g/day in one year?**

- ○ Definitely yes
- ○ Probably yes
- ○ Unsure
- ○ Probably not
- ○ Definitely not

End of Block: Indications. V2

Start of Block: Contraindications

Q1 What is the **minimum** acceptable **Haemogloblin** for native renal biopsy?

- ○ 100 g/l
- ○ 90 g/l
- ○ 80 g/l
- ○ Other (please specify) ____
- ○ No minimum level

Q2 What is the **minimum** acceptable **Platelet count** for native renal biopsy?

- ◯ 150 x 109/l
- ◯ 100 x 109/l
- ◯ 50 x 109/l
- ◯ Other (please specify) ______________________________
- ◯ No minimum level

Q3 What is the **maximum** acceptable **International Normalised Ratio (INR)** for native renal biopsy?

- ◯ 1.2
- ◯ 1.4
- ◯ 1.6
- ◯ Other (please specify) ______________________________
- ◯ No maximum level

Q4 What is the **maximum** acceptable **Systolic Blood Pressure (SBP)** for native renal biopsy?

- ◯ 140 mmHg
- ◯ 160 mmHg
- ◯ 180 mmHg
- ◯ Other (please specify) ______________________________
- ◯ No maximum level

Page Break

Q5 How many **days** should each drug be **stopped for prior** to native renal biopsy?

	0 1 2 3 4 5 6 7 8 9 10
Aspirin ()	
Clopidogrel (Plavix) ()	
Direct oral anticoagulant (DOAC) ()	

Page Break

End of Block: Contraindications

Start of Block: Utility

Q1 *Please rank your opinion on the following statement:*
Renal biopsy is a safe procedure

- ○ Strongly agree
- ○ Somewhat agree
- ○ Neither agree nor disagree
- ○ Somewhat disagree
- ○ Strongly disagree

Q2 *Please rank your opinion on the following statement:*
Renal biopsy helps guide future management

- ○ Strongly agree
- ○ Somewhat agree
- ○ Neither agree nor disagree
- ○ Somewhat disagree
- ○ Strongly disagree

Q3 *Please rank your opinion on the following statement:*
Renal biopsy should be performed by a nephrologist

- ○ Strongly agree
- ○ Somewhat agree
- ○ Neither agree nor disagree
- ○ Somewhat disagree
- ○ Strongly disagree

Page Break

Q4 On a scale from 1 (Strongly Disagree) to 5 (Strongly Agree), to what degree do you feel each factor is a **barrier to renal biopsy?**

	1	2	3	4	5
Time constraints ()					
Location and equipment availability ()					
Risk of complications ()					
Staff availability ()					

End of Block: Utility
